# Carbohydrate-controlled serine protease inhibitor (serpin) production in *Bifidobacterium longum* subsp. *longum*

**DOI:** 10.1038/s41598-021-86740-y

**Published:** 2021-03-31

**Authors:** S. Duboux, M. Golliard, J. A. Muller, G. Bergonzelli, C. J. Bolten, A. Mercenier, M. Kleerebezem

**Affiliations:** 1 Nestlé Research, Lausanne, Switzerland; 2 Host-Microbe Interactomics Group, Wageningen University and Research, De Elst 1, 6708 WD Wageningen, The Netherlands

**Keywords:** Bacterial physiology, Microbiology

## Abstract

The Serine Protease Inhibitor (serpin) protein has been suggested to play a key role in the interaction of bifidobacteria with the host. By inhibiting intestinal serine proteases, it might allow bifidobacteria to reside in specific gut niches. In inflammatory diseases where serine proteases contribute to the innate defense mechanism of the host, serpin may dampen the damaging effects of inflammation. In view of the beneficial roles of this protein, it is important to understand how its production is regulated. Here we demonstrate that *Bifidobacterium longum* NCC 2705 serpin production is tightly regulated by carbohydrates. Galactose and fructose increase the production of this protein while glucose prevents it, suggesting the involvement of catabolite repression. We identified that di- and oligosaccharides containing galactose (GOS) and fructose (FOS) moieties, including the human milk oligosaccharide Lacto-N-tetraose (LNT), are able to activate serpin production. Moreover, we show that the carbohydrate mediated regulation is conserved within *B. longum* subsp. *longum* strains but not in other bifidobacterial taxons harboring the serpin coding gene, highlighting that the serpin regulation circuits are not only species- but also subspecies- specific. Our work demonstrates that environmental conditions can modulate expression of an important effector molecule of *B. longum*, having potential important implications for probiotic manufacturing and supporting the postulated role of serpin in the ability of bifidobacteria to colonize the intestinal tract.

## Introduction

*Bifidobacterium longum* subsp. *longum* is a Gram-positive, high G+C content, anaerobic bacterium that is a member of the phylum Actinobacteria*.* A variety of bifidobacteria species are natural inhabitants of the human gastro-intestinal tract (GIT). In particular, they are dominantly colonizing the infant gut. Bifidobacteria are believed to be acquired by vertical transmission from the mother and their persistence in the infant gut is associated with their saccharolytic activity toward the abundant host and diet-derived glycans present in the infant gut^1^. Human Milk Oligosaccharides (HMOs) are complex carbohydrates found in human milk where they are the third most-abundant solid component^2^. HMOs, as well as lactose, galacto-^3,4^ and fructo-oligosaccharides^5^ in infant formula have been shown to drive *Bifidobacterium* enrichment in the infant gut. In addition, bifidobacteria remain present in the human GIT throughout life and various prebiotic dietary supplements (including galacto- and fructo- oligosaccharides) also enrich the bifidobacterial populations in adulthood. According to recent estimates and depending on the geographical location, *B. longum* subsp. *longum* can make up to 20 % of the *Bifidobacterium* community in the intestine, which can constitute up to 4% of the overall microbiota in adults^1^. Strains belonging to the *Bifidobacterium* genus, including those belonging to *B. longum* subsp. *longum*, are widely used as probiotics and their beneficial effects on specific intestinal and extra-intestinal pathologies have been documented^6^.

The genome of the strain *B. longum* subsp*. longum* NCC 2705 (hereafter *B. longum* NCC 2705), isolated from infant feces, highlighted its particular adaptation to saccharolytic metabolism relevant in the human gut environment, as illustrated by a large repertoire of genes encoding carbohydrate degrading enzymes and import systems^7,8^. The strain also contains different systems enabling it to resist and evolve in the competitive GIT environment. For example, the genome encodes a bile salt hydrolase and the corresponding efflux system conferring bile acid resistance^9^, as well as genes encoding fimbriae that have been shown to drive its *in vitro* adhesion to mucins^10^. This strain is also able to limit growth of the pathogenic *E. coli* O157 *in vivo* through the production of acetate, which depends on the gene encoding the sugar ABC transporter solute-binding protein (*BL0033*)^11^. Moreover, *B. longum* NCC 2705 produces a serine protease inhibitor (serpin) encoded by the *BL0108* gene, which forms covalent products with pancreatic and neutrophil elastases thereby inhibiting their function^12^. This serpin is proposed to play an important role in the colonization of bifidobacteria by protecting them against host-derived proteases and providing them with a survival advantage in the competitive intestinal environment^12,13^. The serpin’s capacity to inhibit the Human Neutrophil Elastase^12^ may also be involved in the immunomodulatory capacities of the strain^14^ as elastase is released by activated neutrophils at the sites of intestinal inflammation^15^. In line with this role in dampening innate immunity, serpin was demonstrated to play a key role in the anti-inflammatory effect of *B. longum* NCC 2705 in a mouse model of gluten sensitivity^16^. Recently, the serpin of the NCC 2705 strain was reported to prevent enteric nerve activation *in vitro*, which suggest a potential role of this protein in pain reduction in Irritable Bowel Syndrome patients^17^.

In view of the hypothesized colonization and bioactive roles of serpin in bifidobacteria, it is important to understand how its production is regulated. Serpin encoding genes have been identified in a limited number of bifidobacterial species, including *B. breve*, *B. longum* subsp. *longum*, *B. longum* subsp*. infantis*, *B. longum* subsp. *suis* and *B. dentium*^13^. However, querying the genomes of a range of recently described bifidobacterial species^18^ identified several additional serpin-encoding genes, suggesting that the function is more widely spread among the members of this genus (Table S1). A previous study failed to detect serpin production by western blot in MRSc grown *B. longum* NCC 2705 and serpin was thus considered to be induced *in vivo* by unknown factors (F. Arigoni, personal communications). Transcriptional regulation studies of the *B. breve* serpin-encoding gene showed that it involves a protease inducible two-component system located next to the serpin encoding operon^19^. This system is absent from *B. longum* subsp *longum* strains and is partially present in strains of *B. longum* subsp. *infantis*. In all studied species, it was shown that the serpin encoding gene is flanked by genes encoding a Lac-I regulator and a membrane-associated protein of unknown function, although the latter gene is not universally conserved^13^. The observed variations in the gene syntenies around the serpin gene suggest that its regulation may not only differ in different species, but may even be subspecies specific^13^. We have here revealed that different carbohydrates control production of serpin, a protein playing a key role in the bioactivity of *B. longum* subsp*. longum.*

## Results

### Development of a sandwich ELISA enabling serpin quantification

**Figure 1:**
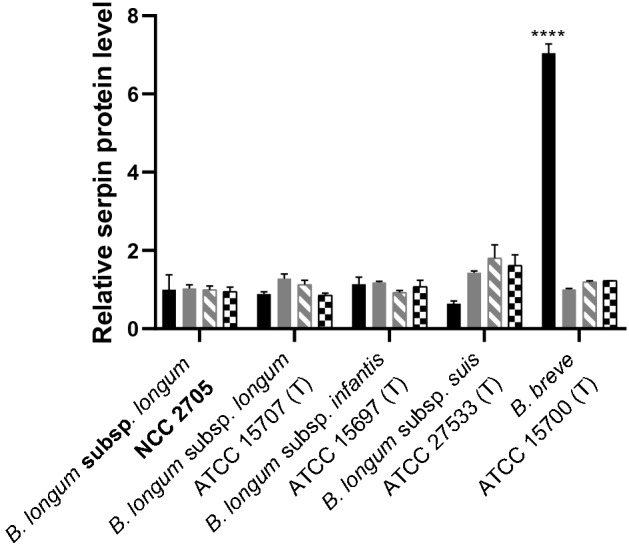
Effect of different proteases (papain [black bars], trypsin [grey bars], chymotrypsin [dashed gray bars] and pig pancreatic elastase [PPE, squared black bars]) on the serpin production levels in bifidobacteria harboring the serpin encoding gene, after 16 h of growth. The studied strains are B. longum subsp. longum NCC 2705, B. longum subsp longum ATCC 15707 (type strain [T]), B. longum subsp. infantis ATCC 15697 (T), B. longum subsp. suis ATCC 27533 (T) and B. breve ATCC 15700 (T). Serpin levels are expressed relative to the control level (including standard deviations) measured in MRS without addition of proteases. Statistical difference relative to the control cells are indicated; ****p < 0.0001.

Polyclonal antibodies obtained by immunizing rabbits with the previously purified *B. longum* NCC 2705 serpin recombinant protein^12^ were used to develop a sandwich ELISA (Figure S1). This method enabled the accurate quantification of the serpin protein in subcellular fractions and crude lysates of *B. longum* NCC 2705, with a limit of quantification (LoQ) of 4 pg/ml of serpin. Moreover, it allowed the detection and quantification of the serpin from *B. breve* ATCC 15700, a protein with only 93% sequence identity compared to the *B. longum* subsp. *longum* serpin^13^ (Figure 1).

### Localization of Serpin in B. longum NCC 2705

**Figure 2:**
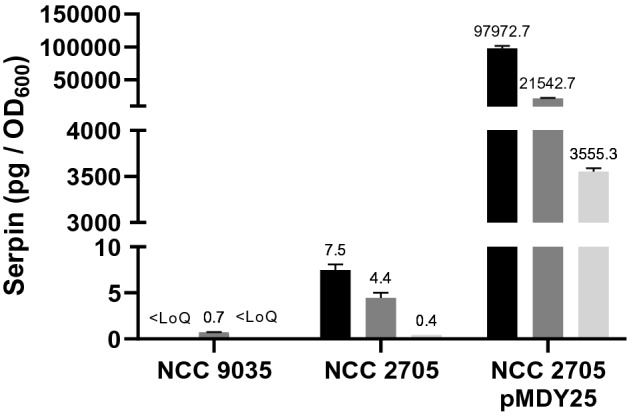
Serpin concentration and localization in extracts of B. longum NCC 2705, its serpin knock-out counterpart (NCC 9035) and the constitutively producing (NCC 2705 harboring pMDY25) recombinant strain. Means and standard deviations are depicted. Black bars represent cytoplasmic fractions, dark grey bars represent cell-wall associated fractions and light grey represent supernatant fractions (i.e. excreted protein). Serpin amounts are reported in picograms (pg) per mg of total protein.

Examination of the *B. longum* NCC 2705 BL0108 serpin protein sequence using Interpro-scan^20^ predicted that the protein contains a N-terminal transmembrane domain^12^. Analysis by the Signal P^21^ package did not predict a signal peptidase cleavage site (Figure S2), suggesting that the protein would be N-terminally anchored by a transmembrane domain. We investigated serpin localization by the comparative analysis of serpin abundance in cell-associated (cytoplasmic, cell-wall) and the spent culture supernatant (secreted protein) fractions (Figure 2). This analysis revealed that both in the wildtype strain *B. longum* NCC 2705 and in its pMDY25-harboring serpin overexpressing derivative^16^, the serpin protein remains associated with the cells and is not released in the environment which in part confirms the predicted localization. The level of serpin produced by the recombinant strain is drastically (> 10,000-fold) higher than the level found in its wildtype counterpart. Importantly, no serpin could be detected in cell-associated or supernatant fractions from strain *B. longum* subsp. *longum* NCC 9035, a genetically engineered serpin null-mutant of NCC 2705^16^ (Figure 2). The minimal background reactivity observed in the cell-wall associated fraction of the NCC 9035, was assumed to be due to minor cross-reactivity of the polyclonal antibody used.

### Evaluation of extracellular proteases as inducing factor for serpin production

*B. longum* subsp. *longum* NCC 2705, *B. longum* subsp. *longum* ATCC 15707 (type strain [T]), *B. longum* subsp. *infantis* ATCC 15697 (T), *B. longum* subsp. *suis* ATCC 27533 (T) and *B. breve* ATCC 15700 (T), all belonging to different non-pathogenic species that encode a serpin orthologue closely related to Bl0108 protein (with % of identity of 99.8, 94.9, 92.7 and 93.3 respectively), were grown in presence of different proteases (papain, trypsin, chymotrypsin and pig pancreatic elastase) previously shown to modulate serpin mRNA levels^13,19^. Cell-exposure to papain induced a 7-fold increase in serpin production in *B. breve* ATCC 15700, which is in agreement with the previously reported increased transcription of the serpin gene in this strain^19^. Conversely, none of the studied proteases led to a significant increase in the levels of cell associated serpin in *B. longum* subsp*. longum, B. longum* subsp *infantis* and *B. longum* subsp. *suis* strains, indicating that serpin production is differentially regulated among these bifidobacterial species (Figure 1).

### Galactose and fructose driven induction of B. longum NCC 2705 serpin is repressed by glucose

**Figure 3:**
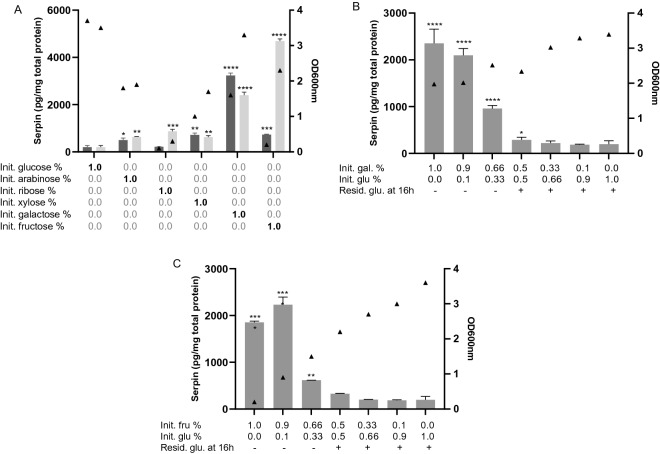
Serpin concentration measured in extracts of B. longum NCC 2705 grown for 24 (dark grey) and 48 h (light grey) on different monosaccharides (**A**). Serpin concentration measured in B. longum NCC 2705 grown for 16 h on different concentrations of glucose and galactose (**B**) and glucose and fructose (**C**). Bars represent serpin quantity normalized by total protein content, means and standard deviations are depicted. Initial carbohydrate concentrations are depicted. Presence of residual glucose in the culture supernatant after 16 h of incubation is indicated (+ /−) (**B**,**C**). Triangles represent growth at the end of the incubation, measured by optical density at 600 nm. *P*-values represent statistical difference to the glucose control group. ****p < 0.0001; ***p < 0.001; **p < 0.01; *p < 0.05.

To test the capacity of different carbohydrates to induce serpin production in *B. longum* NCC 2705 the strain was grown on carbohydrate-free MRSc medium supplemented with 1% (w/v) of different monosaccharides previously shown to support its growth, i.e., glucose, arabinose, ribose, xylose, galactose and fructose^8^. Growth on the majority of the sugars tested led to a significant increase of serpin levels compared to those measured after growth on glucose (Figure 3A). While only a modest increase in serpin production was observed when the strain was grown on the pentoses arabinose, ribose and xylose, growth on hexoses led to strongly increased levels of serpin, i.e., 16- and 24-fold increase after growth on galactose and fructose, respectively. In these experiments all media were inoculated using a glucose grown preculture which explains the delayed growth observed in some of the conditions. The relatively poor overnight growth observed for *B. longum* NCC 2705 on fructose-media is reflecting the slow-adaptation of the strain to the utilization of this sugar. This was further confirmed by extending the incubation on fructose-media to 48 hours and reaching high OD_600_ values (Figure 3A). Nevertheless, despite the relatively poor growth on fructose media in the initial overnight culture, these growth conditions led to a strongly increased level of serpin (Figure 3A,C).

To assess the potential inhibitory effect of glucose, serpin production was next measured after growth of *B. longum* NCC 2705 in media containing different ratios of glucose:galactose (Figure 3B) and glucose:fructose (Figure 3C). In both set of experiments, optical density at 600 nm (OD_600_) and residual glucose levels in the supernatant were measured after 16h of incubation. We observed that the presence of glucose in the medium prevented the galactose- and fructose-mediated induction, which could be indicative of glucose-mediated catabolite repression. Once the glucose was depleted from the medium (Figure 3B,C), the incubation in galactose and fructose media led to induction of serpin production. Notably, the induction of serpin production in fructose media did not appear to depend on growth on this substrate (Figure 3C).

### Galactose induction mechanism is conserved in B. longum subsp. longum strains

**Figure 4:**
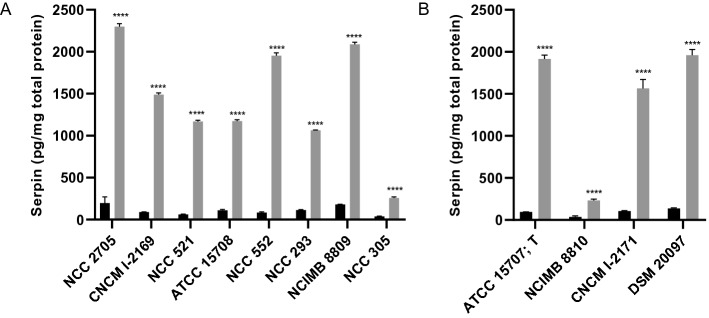
Serpin protein concentration in a set of B. longum subsp. longum strains grown for 16 h on MRSc medium supplemented with 1% glucose (black bars) or 1% galactose (grey bars). Bars represent means and standard deviations are shown. Panel A depicts B. longum subsp. longum strains able to rapidly grow on galactose as a sole carbon source). Panel B displays B. longum subp. longum strains not able to rapidly switch to galactose as a sole carbon source. These strains were grown on a medium supplemented with 1% glucose (black bars) or with a mixture of 0.2% glucose and 0.8% galactose (grey bars). *P*-values represent statistical difference to the glucose control groups. ****p < 0.0001.

Different *B. longum* subsp. *longum* strains (Table S2) were selected to represent the phylogenetic diversity of this subspecies, and these strains were grown on galactose as a sole carbon source for 16h. In these experiments galactose containing media were inoculated with cultures grown in glucose medium. Several strains displayed efficient growth on galactose under these conditions (NCC 2705, CNCM I-2169, NCC 521, ATCC 15708, NCC 552, NCC 293, NCIMB 8809, NCC 305), and in all these cultures a significantly increased level of serpin was detected as compared to their glucose-grown counterparts (Figure 4A). However, galactose did not support growth of all strains within 16h of incubation (ATCC 15707, NCIMB 8810, CNCM I-2171 and DSM 20097), which could reflect strain-specific inability to utilize galactose or a longer lag phase when switching from glucose to galactose. For these strains, a small amount of glucose (0.2%) was added to the galactose-containing (0.8%) medium to allow the production of biomass during the first 16h of incubation, while also including a prolonged period of incubation in galactose containing medium when glucose was depleted. Under these conditions, incubation in galactose containing medium led to increased serpin production (Figure 4B). These results demonstrate that galactose is consistently inducing serpin production in *B. longum* subsp*. longum* strains even in strains that are unable to grow efficiently on this monosaccharide as a sole carbon source.

Similar experiments were performed to evaluate the conservation of the observed serpin regulation in other *Bifidobacterium* species, and/or subspecies of *B. longum*, revealing that galactose-induced serpin production is conserved in *B. longum* subsp. *suis* ATCC 27533 (T) (10.8 fold compared to glucose grown culture) but not in *B. longum* subsp. *infantis* ATCC 15697 (T) or *B. breve* ATCC 15700 (T) (Figure S3). This demonstrates that differential serpin regulation is not only observed when comparing different *Bifidobacterium* species, but also among different *B. longum* subspecies.

### Fructose and galactose containing di- and oligosaccharides induce serpin production

*B. longum* NCC 2705 was grown to stationary phase on MRSc medium supplemented with different galactose or fructose containing disaccharides (lactose, melibiose and saccharose), galacto- or fructo-oligosaccharides (GOS and FOS), or human milk oligosaccharides (HMOs). Serpin production was quantified in crude cell extracts derived from the resulting cultures.

**Figure 5:**
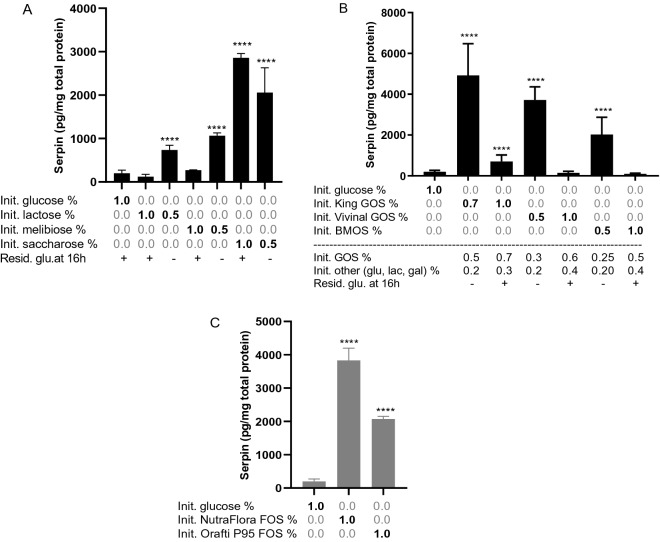
Serpin concentration in extracts of B. longum NCC 2705 grown to the stationary phase (16 h) on MRSc medium, supplemented with the di-saccharides galactose, melibiose and saccharose (panel A), different galacto-oligosaccharides (GOS) (panel B), or different fructo-oligosaccarides (FOS) (panel C). Bars represent means and standard deviations are depicted. Initial carbohydrate concentrations as well as the presence of residual glucose at harvesting are indicated (**A**,**B**). *P*-values represent statistical difference to the glucose control groups. ****p < 0.0001.

Growth on lactose (Gal-β[1→4]-Glc) and melibiose (Gal-α[1→6]-Glc) induced serpin production (respectively 3.7 and 5.2 fold compared to growth on glucose) when supplemented at a concentration of 0.5% (w/v) but not at 1.0 % (w/v). In the latter condition, glucose generated by the hydrolysis of the disaccharides was not depleted at harvesting (16h, Figure 5A). In contrast, saccharose (Glc-α[1→2]-Fru), a fructose-containing disaccharide led to increased levels of serpin irrespective of the concentration used (0.5% or 1.0% supplementation) (Figure 5A).

Growth on different GOS preparations led to a maximal 25-fold induction of serpin in *B. longum* NCC 2705 (Figure 5B). The GOS preparations contain relatively high level of glucose and lactose (King GOS, 28%; Vivinal GOS, 40%; BMOS, 38%). Consequently, also for these substrates the serpin inducing effect was lost at higher supplementation concentrations, correlating with the presence of residual glucose at the time of harvest (16h) (Figure 5B). The FOS preparations tested are purer than GOS and do not contain glucose, explaining why these substrates consistently induced serpin production in *B. longum* NCC 2705 irrespective of the supplementation concentration (Figure 5C).

**Figure 6:**
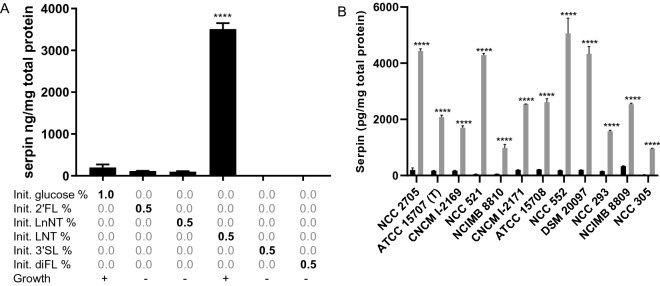
Serpin concentrations in extracts of B. longum NCC 2705 grown to the stationary phase (16 h) on MRSc medium, supplemented with the human milk oligo-saccharides (2′-O-Fucosyllactose [2′FL], Lacto-N-neotetraose [LNnT], Lacto-N-tetraose [LNT], 3′-O-Sialyllactose [3′SL], Difucoxyllactose [DiFL]) (panel A). Different B. longum subsp. longum strains grown to stationary phase(16 h) on MRSc supplemented with 1% glucose (black bars) or 0.5% LNT (grey bars) (panel B). Bars represent means and standard deviations are depicted. *P*-values represent statistical difference to the glucose control groups. ****p < 0.0001.

Finally, *B. longum* NCC 2705 was grown in media containing 0.5% of different HMOs (2’FL, LnNT, LNT, 3’SL and diFL) as sole carbon source. The strain could only grow on Lacto-N-tetraose (LNT), which also led to a 18-fold induction of serpin production as compared to glucose (Figure 6A). LNT was subsequently shown to consistently support the growth and induce serpin production in the different strains representing the phylogenetic diversity of the *B. longum* subsp*. longum* subspecies (Figure 6B).

Taken together these results establish that growth of *B. longum* NCC 2705 (and several other strains of the same subspecies) on galactose and fructose containing di- and oligosaccharides consistently led to increased serpin production. This induction was observed only when the glucose moieties of the galactose containing substrates were completely depleted, which was not the case for the fructose containing carbohydrates.

### AraQ involvement in serpin regulation?

**Figure 7:**
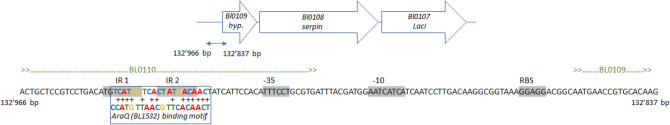
Genetic setup of the serpin operon promoter as previously described by Turroni et al. Inverted repeats (IR 1 and IR 2), -35 and -10 hexamers as well as Ribosome Binding Site (RBS) are depicted in grey. The motif identified using MAST and resembling the previously predicted AraQ motif ahead of the BL1532 gene is depicted with bold colors.

A MAST search using all transcriptional factors predicted to be implicated in the carbohydrate metabolism of *B. longum* NCC 2705 enabled to identify three LacI-type regulator binding sites in the previously predicted promoter region upstream of the *Bl0109* gene^13^. Two binding sites resembling Bl0185 (BL0187) and GosR (BL0258) motifs were located far away from the start of the *Bl0109* gene (respectively 692 and 1107 bp). In contrast, a motif resembling the predicted AraQ (BL1532) binding site was found at a reasonable distance from the serpin operon (94 bp upstream from the Bl0109 start-codon) (Table 1). The resemblance of the potential AraQ binding site is modest when compared to the original BL1532 AraQ binding site but its close localization to the predicted -35 promoter-element supports a role in serpin operon regulation (Figure 7).

**Table 1:** List of transcription factor binding sites predicted by MAST upfront the promoter region of the BL0109 gene.

Regulon name	Regulon type	Original sequence (from RegPrecise)	Target sequence upfront of serpin operon	N° of mismatches	Distance from Bl0109 start (bp)	*p*-value obtained from MAST
AraQ (BL1532)	LacI	CCATGTTAACGTTCACAACT	TCATGGTCACTATGACAACT	6	− 94	3.5E−05
BL0185 (BL0187)	LacI	ATATTGCATCGATGTAAATA	CTATTGCATCGATGCTGATT	5	− 692	6.9E−06
GosR (BL0258)	LacI	TTGGTCAACCGGTGTATCAA	CTGGTCAAGCGTGGTATTAA	5	− 1107	4.1E−06

## Discussion

In this work, we have developed a sensitive ELISA assay which enabled to quantify levels of serpin present in different bifidobacterial species. Using this assay, we could confirm the high levels of serpin produced by the serpin overexpressing pMDY25-harboring derivative of *B. longum* NCC 2705^16^ as well as the lack of serpin detection in the serpin-null mutant *B. longum* NCC 9035 previously used to decipher the role of serpin in a mouse model of celiac disease^16^. Serpin localization experiments using *B. longum* NCC 2705 showed that the protein is retained in the cellular biomass and not secreted to the medium, which confirmed the predicted N-terminal anchoring of the protein in the cell membrane in combination with the lack of a recognizable signal peptidase cleavage site. However, serpin release in the medium was more prominently detected in the culture of the serpin-overproducing *B. longum* NCC 2705 that harbors pMDY25, which is probably reflecting hampered protein biogenesis as a consequence of the very high serpin production in this strain. Nevertheless, the results establish that to asses regulation of serpin production levels by culture conditions, the protein is best quantified by evaluating its specific-level in the cellular biomass and can ignore the very minor amounts that are released into the medium.

Serpin encoding genes have been found to be conserved among a subset of bifidobacterial species^13^. Previous studies have reported that its transcription in *B. breve* is induced by treatment of the cells with proteases, in particular papain, involving a two-component regulatory system, which is not encoded in *B. longum*^13,19^. The observation in *B. breve* is in apparent agreement with the proposed role of serpin in protection of the bacterial cell against the detrimental impact of environmental proteases. Our results confirmed that serpin production is induced by papain treatment in *B. breve* and established that a similar treatment with papain or other proteases failed to induce it in various *B. longum* subspecies (*longum*, *infantis*, and *suis*). Notably, we could establish that serpin production in *B. longum* subsp. *longum* and *B. longum* subsp. *suis* is controlled by the carbon source in the culture medium, identifying galactose and fructose as potent inducers*.* Furthermore, our results suggest that import of these carbohydrates in the cell is essential for their effect on serpin production, even though their metabolization appears not required (Figures 3C, 4B). None of the carbon sources tested was able to modulate serpin levels in *B. longum* subsp*. infantis.* This highlights that diverse mechanisms regulate production of serpin in different bifidobacterial species (e.g. *B. breve* versus *B. longum*), but also in different subspecies. The regulation of serpin production by carbon sources in specific *B. longum* subspecies does not have an obvious relation with the anti-protease function of serpin. This may suggest that an additional functional role of this protein remains to be deciphered.

The data we present suggest the involvement of catabolite repression in the control of serpin production. Growth of *B. longum* on the pentoses arabinose, ribose and xylose led to a modest but statistically significant increase of serpin production compared to glucose, which may be due to relieved glucose catabolite repression. This is in sharp contrast to the prominent serpin-inducing capacity of the hexoses galactose and fructose. Even though both pentoses and hexoses are metabolized through the so called “bifid-shunt”, their metabolism is markedly different; pentoses are entering the energy generating phosphoketolase-dependent pathway as glyceraldehyde-3-phosphate, while hexoses are converted by phosphoglucomutase to glucose-6-phosphate and fructose-6-phosphate^22^. Metabolic intermediates of the “bifid-shunt” may play an important role in the regulation of serpin and would deserve to be further studied in this context.

**Figure 8:**
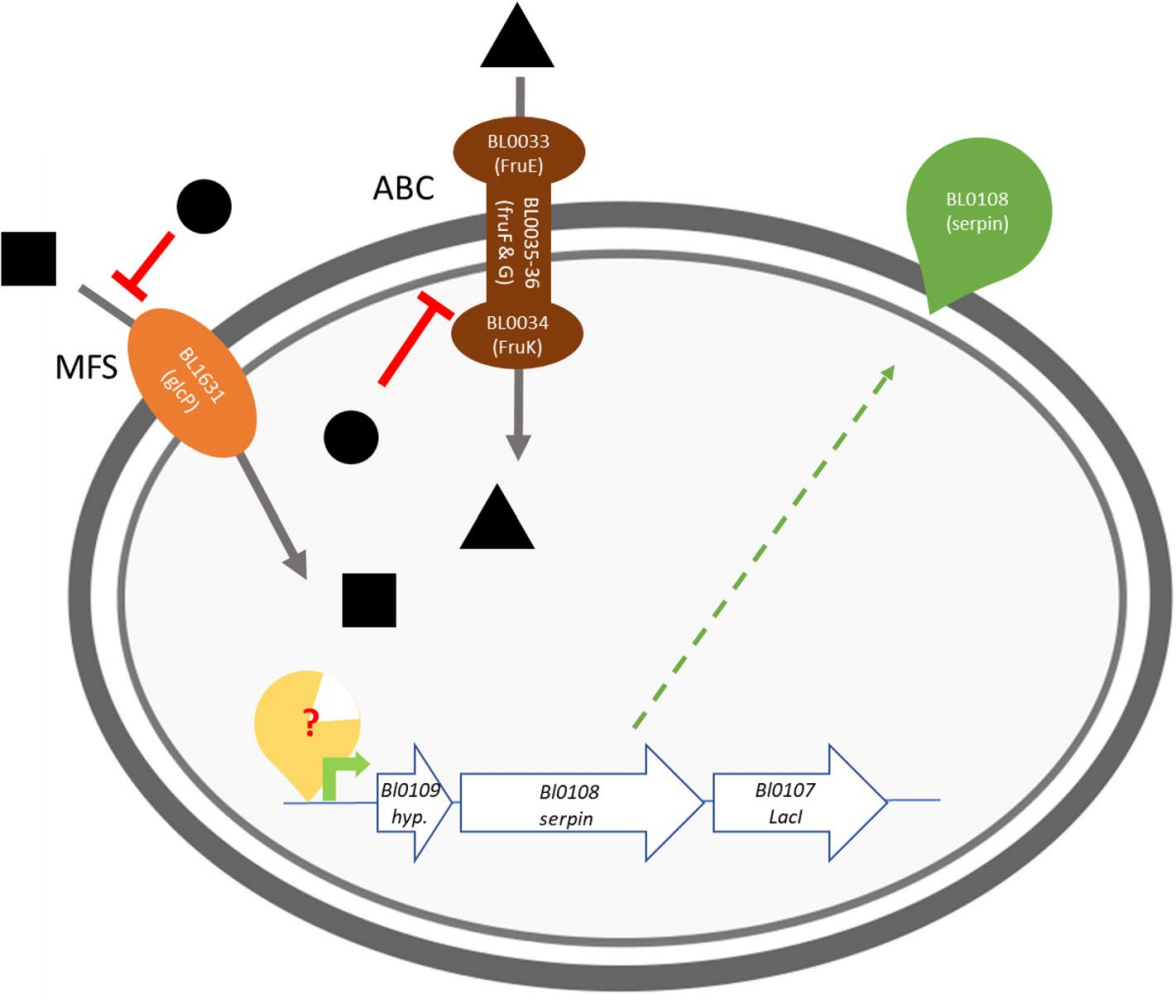
Proposed schematic representation of serpin induction by galactose or fructose and its repression by glucose. Presence of glucose (black circles) inhibits transport of galactose (black squares), as it is known to have a higher affinity to the Major Facilitator Superfamily (MFS) transport protein glcP protein (BL1631). As well, presence of glucose in the cell has been previously shown to inhibit the production of the ATP-binding cassette transporters (ABC) encoded by the genes located in the operon BL0033-BL0036 and responsible of fructose (black triangles) import. The exact regulatory genetic mechanism enabling serpin production remains to be elucidated.

Although the precise molecular mechanisms involved in serpin regulation in *B. longum* NCC 2705 remains to be elucidated, we could demonstrate that galactose- and fructose-mediated serpin induction is prevented by presence of glucose in the environment. In *B. longum* NCC 2705, the glucose/mannose transporter protein (encoded by BL1631; glcP) is involved in galactose import^8^. This protein was demonstrated to have the highest specificity for glucose followed by mannose and galactose^23^, which could explain why glucose depletion is required to enable the subsequent import of galactose that once internalized could play a role in the intracellular activation mechanism of serpin production. In the same strain, an ATP-binding cassette transporter encoded by the operon Bl0033-0036 (*fruEKFG*) mediates fructose import. Previous studies have shown that the synthesis of the substrate binding protein (FruE) is strongly suppressed by the presence of glucose^24^, suggesting that the *fru* operon is suppressed by the presence of glucose in the medium. Thereby, the presence of glucose in the medium could inhibit fructose-import, and assuming that internalized fructose (analogous to internalized galactose) could play a role in in the intracellular serpin production-activating regulatory mechanism provides a rational explanation of glucose suppression of fructose-mediated activation of serpin production (Figure 8)*.*

These results resemble typical manifestations of glucose mediated catabolite repression, which is well described in many bacteria^25^ and for which a few examples have been described in bifidobacteria^23,26,27^. In most Gram positive bacteria, catabolite repression is controlled by the complex formed by the histidine phosphocarrier protein (HPr) and catabolite control protein A (CcpA), a LacI-type regulator that can bind to specific DNA motifs (so-called *cre*-elements) and modify transcription of downstream genes^28^. At present, the closest relatives of the *Bacillus subtilis* CcpA in *B. longum* NCC 2705 are annotated as LacI-type regulators, and their homology with CcpA is quite low (24-32% identity). Bifidobacteria encode a large number of LacI-type regulators known to commonly regulate carbohydrate metabolism. For example, *B. longum* NCC 2705 harbors 16 genes encoding LacI-regulators according to the RegPrecise database^29^. Most of these LacI-type regulators are foreseen to act locally by regulating genes located in their vicinity. This is exemplified by the LacI encoding gene *BL0107* that is genetically linked to the serpin encoding gene and is predicted to regulate the closely located sucrose utilization operon that encodes a sucrose permease (BL0106) and a sucrase (BL0105). Recently, two LacI-type transcriptional regulators, AraQ and MalR1, were demonstrated to control a large set of genes spread over the genome of *B. breve* UCC 2003 including genes involved in the”bifid-shunt” as well as transcription factors, and uptake and metabolism of various carbohydrates^30,31^. Using the online Motif Alignment & Search Tool (MAST)^32^, we did not detect MalR1 binding sites, but did identify an possible AraQ binding motif in the promoter region upstream of the serpin-encoding operon (i.e., upstream of *BL0109*). The resemblance of the AraQ cis-element and the sequence found in the serpin promoter region is modest, but in our opinion warrants further experimental investigation to investigate the proposed involvement of AraQ in serpin regulation in *B. longum* NCC2705 and other members of the same (sub-)species.

The galactose containing di- and oligosaccharides (i.e. lactose, melibiose and galacto-oligosaccharides [GOS]) as well as the fructose containing di- and oligosaccharides (i.e. saccharose and fructo-oligosaccharides [FOS]) induce serpin production. Importantly, the presence of glucose in the oligosaccharides prevented galactose induction unless the glucose moiety was completely consumed. GOS are produced using enzymatic synthesis from lactose^33,34^ and usually contain high levels of free lactose and glucose that we demonstrated to suppress serpin production. Conversely, FOS are commonly produced by hydrolysis of long chain inulin and therefore have a very low glucose content^34^. Intriguingly, glucose repression was not observed when cells were grown on saccharose, although this disaccharide is composed of a glucose and a fructose moiety. In contrast to lactose, hydrolysis of sucrose by sucrose phosphorylase (*BL0536*; EC 2.4.1.7) results in the formation of fructose and glucose-1-phosphate in the cell. Intriguingly, specific regulatory roles have been suggested for intracellular glucose-1-phosphate, including its proposed role in the regulation of virulence genes in *Listeria monocytogenes*^35^.

Lacto-N-tetraose (LNT) contains two galactose, one glucose and one N-Acetyl-galactosamine residues^2^, and hence was tested for its capacity to induce serpin. At an initial concentration of 0.5 % in the culture medium, LNT supported growth and enabled production of serpin in all tested *B. longum* subsp. *longum* strains, indicating a high conservation of the induction mechanism. LNT is likely imported as a tetra saccharide, as the *B. longum* subsp. *longum* strains lacks the gene encoding an extracellular lacto-N-biosidase that would be required for this reaction^36^. Assuming that LNT is imported as a tetra saccharide into the cell, the enzymes involved in its intracellular hydrolysis remain to be identified. However, removal of the terminal glucose moiety may be catalyzed by β-galactosidase. The proposed glucose-mediated catabolite repression may thereby also be transiently activated during LNT metabolization but may have remained unobserved in our experiments due to the low concentration of LNT (0.5%) used. The remaining hydrolysis product (Galβ1–3GlcNAcβ1–3Galβ1) is likely cleaved by the lacto-N-biose phosphorylase (encoded by the gene *BL1641*) into 2 molecules of galactose and one N-acetyl-galactosamine (39). Although we deciphered the role of glucose and galactose in serpin regulation, a possible involvement of N-acetyl-galactosamine remains to be clarified.

Today, even if FOS and GOS are not the oligosaccharides present in human milk, they are commonly used in infant formula as an affordable alternative to HMOs (including lacto-N-tetraose (LNT)^37^) with the purpose to drive *Bifidobacterium* enrichment in the infant gut^3–5^. Our work suggests that these oligosaccharides could have an impact that goes beyond bifidobacterial growth stimulation, by modifying the *in situ* expression of a specific molecule (i.e., serpin), which has been proposed to play a role in colonization and immunomodulatory properties of *B .longum* subsp. *longum* strains.

## Methods

### Strains and growth conditions

All strains used in this study (Table S2) were obtained from the Nestle Culture Collection (NCC) (Nestlé Research, Lausanne, Switzerland). Growth was routinely performed using de Man, de Rogosa, Sharpe medium (BD Difco, Franklin Lakes, USA) supplemented with 0.05 % of cysteine (MRSc), at 37 °C in anaerobiosis without agitation. Supplementation of the media with 200 µg/ml of spectinomycin was applied for the serpin overexpressing recombinant *B. longum* NCC 2705 that harbors pMDY25^16^. Where relevant, protease addition rates in the medium were the same than previously described^13^, namely 0.5 mg/ml papain, 0.1 mg/ml trypsin, 0.16 mg/ml chymotrypsin (Sigma-Aldrich Chemie GmbH, Buchs, Switzerland) and 1 mg/ml porcine pancreatic elastase (PPE; MP Biomedicals SARL, Illkirch-Graffenstaden, France). Carbohydrate stock solutions were all prepared at 100 g/L in water and filtered sterilized (glucose, arabinose, ribose, xylose, galactose, fructose, lactose, melibiose, saccharose; Sigma-Aldrich Chemie GmbH), galacto-oligosaccharide (GOS) (King GOS [King-Prebiotics, Yunfu City, China]; Vivinal GOS [FrieslandCampina DOMO Amersfoort, The Netherlands]); bovine milk oligosaccharides BMOS [Société des Produits Nestlé, Vevey, Switzerland^4^], fructo-oligosaccharides (FOS) (NutraFlora FOS [Ingredion Korea Inc, Gyunggi-do, Korea]; Orafti P95 [BENEO GmbH, Mannheim, Germany]) and human milk oligosaccharides (HMOs) (2'-O-Fucosyllactose (2'FL), Lacto-N-tetraose (LNT), Lacto-N-neotetraose (LNnT), 3'-O-Sialyllactose (3'SL), Difucoxyllactose (DiFL) [Glycom A/S, Lyngby, Denmark]). These carbohydrates were added to a MRSc-based medium containing no carbohydrates (10 g/L bacto proteose peptone, 3, 5 g/L bacto yeast extract, 1 g/L Tween 80 [Chemie Brunschwig, Basel, Switzerland]; 2 g/L di-ammonium hydrogen citrate, 5 g/L sodium acetate, 0.1 g/L magnesium sulphate, 0.05 g/L manganese sulfate, 2 g/L di-sodium phosphate, 0.5 g/L cysteine [Sigma-Aldrich Chemie GmbH]). Two (2) % rate inoculum from an MRSc overnight culture was applied for all growth experiments, which were performed in a BioLector microbioreactor system (m2p-labs GmbH, Baesweiler, Germany), using 48 flowerplate inserted in an anaerobic chamber for 16 to 48h (2ml volume per well, agitation at 600 rpm, CO_2_ atmosphere, 37 °C). Growth was followed over time by continuous measurement of the scattered light at 620 nm and levels of residual glucose in the culture were determined using the MQuant kit (Sigma-Aldrich Chemie GmbH) according to the manufacturer’s protocol.

### Subcellular fractions preparation, crude protein extraction and total protein quantification

To determine the subcellular localization of the serpin, cells were grown to stationary phase (16h) and a cell amount corresponding to 12 units of OD_600_ was harvested by centrifugation (3500 g, 2 min, 4 °C). The supernatant was filtered through a 0.2 µm pore size filter (Sigma-Aldrich Chemie GmbH), and the filtrate was precipitated with an equal volume of ice-cold acetone and placed on ice for 20 minutes prior to centrifugation (12,000 g, 10 min, 4 °C). The obtained pellet was dissolved with 960 µl of 50mM NaOH (i.e., supernatant fraction). The bacterial pellet was washed once with one volume of ice-cold PBS and resuspended in 960 µl of PBS containing 0.1 mg/ml DNAse I, 20 μg/ml Rnase A and SigmaFast Protease Inhibitor (Sigma-Aldrich Chemie GmbH; 1 tablet per 100 ml of solution). The obtained solution was incubated at 37 °C for 30 minutes and was subsequently lysed by bead-beating using a FastPrep-24 (MP Biomedicals SARL), 3 times for 1 minute at 4 m/s, with 2 minutes cooling on ice in between. The obtained crude cell lysate was separated in cytoplasmic (soluble) and crude cell wall (pellet) fractions by centrifugation (12,000 g, 10 min, 4 °C). The cell wall fraction pellet was further rinsed twice and resuspended in 960 µl of PBS containing proteinase inhibitor, which was tested to not interfere with the developed ELISA. All fractions were analyzed for serpin content (see below) and obtained values were normalized based on culture optical density, measured at 600 nm (OD_600_).

For all other analyses, stationary phase bacterial cultures (after 16h of growth, unless stated otherwise) were harvested by centrifugation (3500 g, 2 min, 4 °C). In order to quantify residual glucose content, supernatants were filter sterilized using a 0.45 µm filter and stored at − 20 °C. Bacterial pellets were washed with one volume of Dulbecco’s Phosphate Buffer (PBS; Sigma-Aldrich Chemie GmbH) and resuspended in 600 µl of PBS containing Halt Protease Inhibitor (Sigma-Aldrich Chemie GmbH). Bacteria were subsequently lysed by bead-beating using a FastPrep-24 (see above; MP Biomedicals SARL). Crude lysates were used as such, containing both soluble and non-soluble fractions. Total protein content was determined using the Pierce BCA protein Assay kit (Thermo Fisher Scientific AG, Basel, Switzerland).

### Serpin protein quantification by sandwich ELISA

All anti-serpin rabbit polyclonal antibodies used in this work were obtained from Proteogenix (Schiltigheim, France) and using a purified *B. longum* NCC 2705 recombinant serpin produced in *Escherichia coli*^12^. For the sandwich ELISA, 96 well plates (Nunc MaxiSorp [Thermo Fisher Scientific AG]) were used. Coating was performed for 16h at 4 °C using 100 µl per well of a 0.2 M sodium carbonate/biocarbonate solution at pH 9.4 containing 250 ng/ml of primary anti-serpin antibodies. In between each step, plates were washed 3 times using 300 µl of wash buffer (WB; PBS to which 0.05% of Tween 20 was added). Blocking was performed for 1h at room temperature using 300 µl of blocking buffer (BB; WB to which 2% of Bovine Serum Albumin [BSA; Sigma-Aldrich Chemie GmbH] was added). The standard (purified recombinant serpin^12^) and the different samples were diluted in a final volume of 100 µl of BB and incubated for 2h at room temperature. Secondary biotinylated anti-serpin antibody (250 ng/ml in 100 µl of WB containing 0.2% BSA) was added and incubated for 1h at room temperature, followed by the addition of 100 µl of the enzyme conjugate (HRP Pierce, Thermo Fisher Scientific AG) (10 µg/ml in WB containing 0.2% BSA) and continuing incubation for 1h at room temperature. Subsequently, repeated (6) washes using 300 µl of WB were performed to remove background reactivity. Detection was performed by adding 100 µl per well of HRP substrate (1-step Ultra TMB ELISA, ThermoFisher Scientific AG) and 15 minutes incubation at room temperature. The reaction was terminated by the addition of 100 µl of 2M sulfuric acid and final optical density at 450 nm was measured in a Varioskan spectrophotometer (Thermo Fisher Scientific AG).

Standard curves were obtained following the sigmoidal 4 parameter logistic regression (4PL) method and concentrations of serpin in individual samples were calculated using the following formula:

$$ y = \frac{A - D}{{\left( {1 + \left( \frac{x}{C} \right)B} \right) + D}} $$

A: Minimal asymptote ($y$); B: Slope; C: Inflection point ($x$); D: Maximal asymptote ($y$).

### Statistical analysis

All data obtained for *B. longum* NCC 2705 grown in either MRSc or MRSc + 1% glucose were pooled—(n=36) and were analyzed for normality using D’Agostino and Pearson algorithm (data not shown). This data pool was used as control group in every analysis. Data were log transformed to compensate for potential heteroscedasticity and one-way ANOVA was performed on every dataset. *P*-values were obtained by performing a Turkey’s multiple comparison with an alpha level of 0.05.

### Bioinformatic analysis

All gene and protein sequences of *B. longum* NCC 2705 referred to in this work were obtained from the National Center for Biotechnology Information (NCBI; NC_004307.2). Operon prediction was obtained from the Prokaryotic Operon Database (ProOpDB)^38^. Functional annotation of the *B. longum* NCC 2705 serpin protein encoded by the *BL0108* gene was performed using Interpro-scan^20^ and signal peptide prediction was performed using Signal P^21^.

To shed light on the genetic mechanism involved in regulation of serpin production, all bindings sites of transcriptional factors known to be implicated in carbohydrate metabolism regulation were retrieved from the RegPrecise database^29^. These sequences were used as input in the “Motif Alignment and Search Tool” (MAST)^32^, to identify resembling motifs in the region upstream of the *Bl0109* gene (genome positions 132852 to 134216; reverse sequenced). Obtained candidate motif-homologues were subsequently mapped relative to the serpin promoter elements (i.e., the -35 and -10 regions) that were previously described by Turroni et al.^13^.

## Supplementary Information

Supplementary Information.
