# Supplementary material for: Carbohydrate-controlled serine protease inhibitor (serpin) production in Bifidobacterium longum subsp. longum
Source: Sci Rep. 2021 Mar 31;11:7236. doi: 10.1038/s41598-021-86740-y (PMC8012564; doi:10.1038/s41598-021-86740-y)
Supplement: Supplementary file 1 — Supplementary Information. [file 41598_2021_86740_MOESM1_ESM.docx]

**Carbohydrate-controlled serine protease inhibitor (serpin) production in *Bifidobacterium longum* subsp. *longum***

S. Duboux* ^1,2^, M. Golliard ^1^, J.A. Muller ^1^, G. Bergonzelli ^1^, C.J. Bolten ^1^, A. Mercenier ^2^ and M. Kleerebezem* ^2^

^1^ Nestlé Research, Lausanne, Switzerland;

^2^ Host-Microbe Interactomics Group, Wageningen University & Research, De Elst 1, 6708WD Wageningen, The Netherlands

**Supplementary data**

Figure S 1: Standard curve of the developed sandwich ELISA obtained using pure recombinant serpin protein. Nonlinear regression is obtained using the sigmoidal 4 parameter logistic regression (4PL) method.


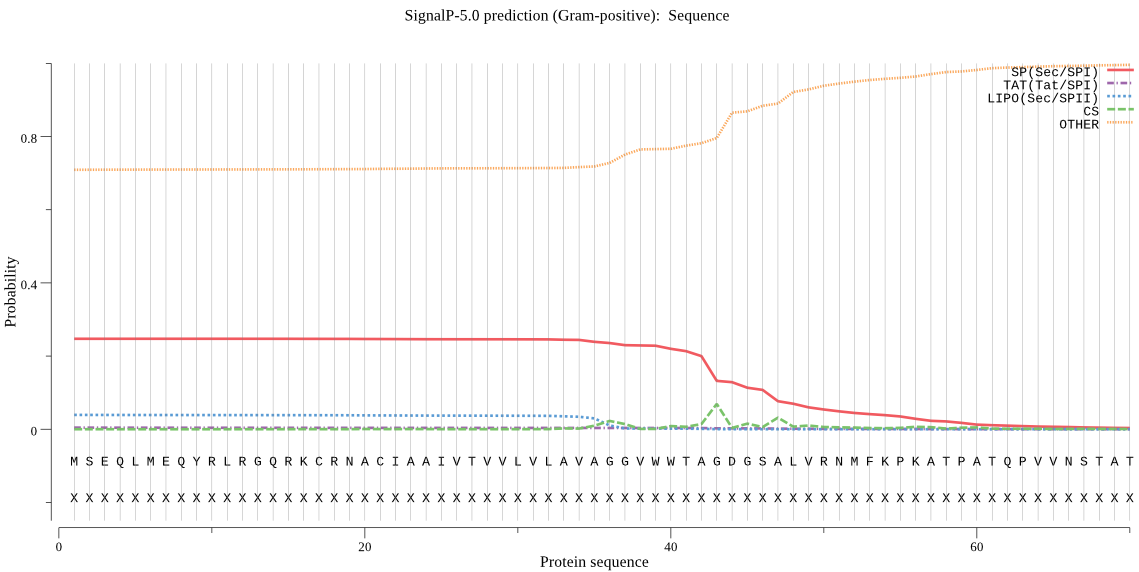


Figure S 2: Bl0108 signal peptide & cleavage site prediction using Signal P 5.0. Graphical representation shows that the protein has a low probability of being cleaved (SP; TAT, LIPO, CS), and a high probability of being non-cleaved .

Figure S 3: Serpin levels in extracts of different *Bifidobacterium* species (harboring the serpin encoding genes) grown until stationary phase (16h) on 1% glucose (black bars), 1% galactose (grey bars)) or 1% glucose in presence of 0.5 mg/ml of papain (dashed grey bars). Bars represent means and standard deviations are depicted. P-values represent statistical difference to each glucose control group. **** p<0.0001; ** p<0.01; * p<0.05

Table S 1: Distribution of Bifidobacterium species harboring Bl0108 serpin homologous proteins identified in RefSeq Bifidobacterial genomes by BLASTP. Unclassified genomes were excluded from the list.

| **Sepcies level taxonomy** | Number of hits | Number of organisms | Best score |
| --- | --- | --- | --- |
| **Bifidobacterium longum** | 97 | 1 | 962 |
| **Bifidobacterium breve** | 32 | 1 | 904 |
| **Bifidobacterium scardovii** | 2 | 1 | 787 |
| **Bifidobacterium callitrichidarum** | 1 | 1 | 566 |
| **Bifidobacterium scaligerum** | 1 | 1 | 537 |
| **Bifidobacterium myosotis** | 2 | 1 | 535 |
| **Bifidobacterium felsineum** | 1 | 1 | 531 |
| **Bifidobacterium imperatoris** | 1 | 1 | 529 |
| **Bifidobacterium saguini** | 1 | 1 | 523 |
| **Bifidobacterium goeldii** | 1 | 1 | 481 |
| **Bifidobacterium stellenboschense** | 1 | 1 | 402 |
| **Bifidobacterium lemurum** | 1 | 1 | 389 |
| **Bifidobacterium biavatii** | 1 | 1 | 360 |
| **Bifidobacterium dentium** | 6 | 1 | 345 |
| **Bifidobacterium moukalabense** | 5 | 1 | 302 |
| **Bifidobacterium pseudolongum** | 27 | 1 | 291 |
| **Bifidobacterium adolescentis** | 2 | 1 | 252 |
| **Bifidobacterium angulatum** | 1 | 1 | 245 |
| **Bifidobacterium choerinum** | 3 | 1 | 222 |
| **Bifidobacterium vansinderenii** | 1 | 1 | 220 |
| **Bifidobacterium callimiconis** | 1 | 1 | 216 |
| **Bifidobacterium simiarum** | 1 | 1 | 214 |
| **Bifidobacterium criceti** | 2 | 1 | 203 |

Table S 2: List of strains used in this work.

| **Strain** | Taxonomy | Origin | Ref |
| --- | --- | --- | --- |
| **NCC 2705 (CNCM I-2618)** | *B. longum subsp. longum* | *Infant isolate* |  |
| **NCC 9035** | *B. longum subsp. longum* | Bl0108 serpin knock-out derivative of NCC 2705 | McCarville et al. 2017 |
| **NCC 2705 with pMDY25** | *B. longum subsp. longum* | pMDY25 harboring derivative of NCC 2705, Bl0108 serpin constitutive over expression strain | McCarville et al. 2017 |
| **ATCC 15707 (T)** | *B. longum subsp. longum* | ATCC (typestrain) |  |
| **ATCC 15697 (T)** | *B. longum subsp. Infantis* | ATCC (typestrain) |  |
| **ATCC 27533 (T)** | *B. longum subsp. suis* | ATCC (typestrain) |  |
| **ATCC 15700 (T)** | *B. breve* | ATCC (typestrain) |  |
| **CNCM I-2169** | *B. longum subsp. longum* | *Infant isolate* |  |
| **NCC 521** | *B. longum subsp. longum* | *Adult isolate* |  |
| **ATCC 15708** | *B. longum subsp. longum* | *ATCC* |  |
| **NCC 552** | *B. longum subsp. longum* | *Adult isolate* |  |
| **NCC 293** | *B. longum subsp. longum* | *Adult isolate* |  |
| **NCC 305** | *B. longum subsp. longum* | *Infant isolate* |  |
| **NCIMB 8809** | *B. longum subsp. longum* | *NCIMB* |  |
| **CNCM I-2170** | *B. longum subsp. longum* | *Infant isolate* |  |
| **NCIMB 8810** | *B. longum subsp. longum* | *NCIMB* |  |
| **DSM 20097** | *B. longum subsp. longum* | *DSMZ* |  |
